# Relaxation Music in Broiler Chicken Production: The Effect of Ambient Music on Pectoral Muscle Quality

**DOI:** 10.3390/ani16081155

**Published:** 2026-04-10

**Authors:** Patrycja Ciborowska, Damian Bień, Anna Zalewska, Jakub Urban, Arkadiusz Matuszewski, Paweł Solarczyk, Karwan Yaseen Kareem, Marta Gajewska, Justyna Więcek, Monika Michalczuk

**Affiliations:** 1 Department of Animal Breeding and Nutrition, Institute of Animal Sciences, Warsaw University of Life Sciences-SGGW, Ciszewskiego 8, 02-786 Warsaw, Poland; anna_zalewska1@sggw.edu.pl (A.Z.); jakub_urban1@sggw.edu.pl (J.U.); pawel_solarczyk@sggw.edu.pl (P.S.); justyna_wiecek@sggw.edu.pl (J.W.); 2 Department of Animal Environment Biology, Institute of Animal Sciences, Warsaw University of Life Sciences-SGGW, Ciszewskiego 8, 02-786 Warsaw, Poland; arkadiusz_matuszewski1@sggw.edu.pl; 3 Department of Animal Production and Health, Salahaddin University-Erbil, Kirkuk Road, Erbil 44002, Iraq; karwan.kareem@su.edu.krd; 4 Department of Animal Genetics and Conservation, Institute of Animal Sciences, Warsaw University of Life Sciences-SGGW, Ciszewskiego 8, 02-786 Warsaw, Poland; marta_gajewska@sggw.edu.pl

**Keywords:** relaxing music, broiler chickens, pectoral muscle quality, stress reduction

## Abstract

Ross 308 chickens were randomly divided into 2 groups of 600 birds each: a control group (C) and an experimental group (M). The birds were reared for 42 days in accordance with the flock management guidelines. Group M was exposed to music for 2 h/day and for 30 min before slaughter (~70 dB). After slaughter, the carcasses were cooled, and after 24 h, the pectoral muscles were collected for further physicochemical analyses. The study results revealed a lower value of drip loss in the pectoral muscles of the chickens from group M than in those from group C (*p* ≤ 0.01). Moreover, the muscles from group M chickens had higher pH values at 15 min, 1 h, and 12 h (*p* ≤ 0.01) and also at 4 h (*p* ≤ 0.05) post-mortem. Pectoral muscles of group M chickens also showed lower L* and b* color parameters (*p* ≤ 0.01), collagen content (*p* ≤ 0.05), and GSH concentration (*p* ≤ 0.01), compared to control birds. Exposure to ambient relaxation music in the scheme used in the study may be an effective form of environmental enrichment for broiler chickens, leading to physicochemical changes in their pectoral muscles consistent with potentially lower pre-slaughter stress.

## Simple Summary

Enriching the environment of farm animals with music is increasingly being studied for stress reduction and potential improvements in the quality of animal-derived food products. It is a relatively inexpensive and simple means to positively influence the behavior and welfare of animals, including chickens. The aim of this study was to assess the effect of ambient relaxation music on the quality of the pectoral muscle of broiler chickens. The results indicate that the track used in the experiment improved post-mortem pectoral muscle stability and had beneficial effects on several meat quality characteristics, including: drip loss, pH, color parameters, collagen content, and GSH concentration. These effects may be due to stress mitigation during rearing, as reflected by a consistent pattern of changes aligned with the assumption that relaxing music beneficially affects broiler chickens.

## 1. Introduction

In 2023, the total global population surpassed 8 billion people, representing an increase of 1 billion people over the past decade [1,2]. A larger population translates into greater consumption of animal-derived products, which constitute one of the key food resources, accounting for approximately one-third of the protein intake by the global population [3]. The pressure faced by the livestock sector makes it challenging to meet such high demand while simultaneously ensuring environmental sustainability. Livestock production conducted in a sustainable manner, i.e., combining intensive and extensive systems, is unlikely to provide a sufficient quantity of animal-based products given the projected global population of 9 billion by 2030 [4]. Poultry production, accounting for almost 40% of the total animal production, plays a key role in meeting population’s protein needs [5]. Over the past decades, this sector has undergone significant changes in terms of production systems, with particular emphasis on environmental and social aspects. However, requirements continue to grow, forcing a constant search for methods to address issues such as product safety, the impact of production on the environment, and animal welfare [6,7]. In light of this, it is important to reduce the stressors to which chickens are exposed during rearing, such as overcrowding, inappropriate temperature, noise, fear, or pain [8,9]. Stress factors contribute to the depletion of the body’s natural antioxidants. This is particularly important before slaughter, as stressors upset the homeostasis of bird cells, triggering physiological and biochemical changes that contribute to meat quality deterioration and a decrease in meat production [10]. The disadvantages resulting from these disorders are reflected in changes in the physicochemical properties of meat, such as color, water-binding capacity, and pH [11]. Acute or short-term stress promotes the formation of PSE (pale, soft, exudative) meat, whereas long-term, chronic stress contributes to the formation of DFD (dark, firm, dry) meat [12]. The latter type of meat has poor technological properties and is more prone to spoilage than meat with normal parameters [13]. It is, therefore, important to reduce stressors or mitigate their impact on chickens. Today, stress management includes various strategies, such as administration of phytogenic feed additives, the use of specific lighting systems, litter changes, climate control, and the introduction of environmental enrichments, such as music [14,15].

The positive impact of selected music tracks or genres on the behavior and welfare of farm animals is well documented. This is particularly evident in experiments involving dairy cows and pigs, although ample literature works have also reported similar beneficial effects of music, including classical music [14,16,17], and ambient relaxation music [18], on these parameters in broiler chickens. The musical piece used in our experiment—“Weightless” by Marconi Union—has been recognized as an effective tool for reducing acute stress in humans. Among other things, it caused a 36.09% decrease in skin conductance and was also proven to offer an alternative to antianxiety medication [19,20]. It is assumed that this music piece may potentially have a similar effect on birds.

An additional aspect of enriching the environment of farm animals with appropriately selected music is its impact on production parameters. Cows exposed to classical music showed an increase in milk yield. Similarly, sows that were played this type of music during pregnancy, farrowing, and lactation raised a greater number of piglets compared to those not exposed to music [21,22]. In broiler chickens, exposure to classical music was reported to reduce the feed conversion ratio (FCR), thereby providing economic benefits [23]. Moreover, Indian classical music (derived from Indian Ragas) has been shown to mitigate the adverse effects of excessive stocking density in broiler chickens, while also increasing their daily body weight gains and a* color parameter (responsible for meat redness) of their pectoral muscle [24].

In light of existing studies confirming the positive impact of environmental enrichment with music on farm animals, it is noteworthy that the sounds of certain musical tracks can also become a stress factor. The use of loud, fast-paced music (e.g., rock ‘n’ roll music) played at high volume results in poorer rearing results of broiler chickens or fattening pigs, e.g., lower body weight gains or increased feed intake [25,26]. Some studies have also indicated that an acoustic stimulus with these parameters may indirectly influence the development of inflammation in the body [27]. However, the available literature does not provide a clear answer as to the mechanism by which the sounds of selected musical pieces affect animal organisms.

In our previous study, broiler chickens were exposed to ambient relaxation music (“Weightless” by Marconi Union). The results indicated a significant effect of this environmental stimulus on selected production outcomes, including final body weight and feed conversion ratio (FCR). The acoustically stimulated group also showed improved welfare indicators (gait score, footpad dermatitis, hock burn, and plumage cleanliness) and a lower concentration of a stress-related hormone in excreta compared with the control group not exposed to the auditory stimulus. Moreover, a potential effect of the music on physiological levels of selected blood parameters was suggested [18].

The available literature is dominated by studies focusing on the effects of music on the behavior, welfare, and production parameters of farm animals, while few publications so far have discussed its indirect impact on meat quality and post-slaughter changes [24,28,29]. The aim of the study was, therefore, to evaluate the effect of exposing broiler chickens to “Weightless” by Marconi Union on selected physicochemical parameters of their pectoral muscle.

## 2. Materials and Methods

### 2.1. Animals and Music Playback

Prior to conducting the experiment, a written statement was obtained from the Second Local Ethical Committee for Animal Experiments at the Warsaw University of Life Sciences, indicating that the research described in the application did not require the consent of the Ethical Committee (Justification dated 24 November 2021). Male Ross 308 chicks, which constituted the research material, were randomly divided into two equal groups of 600 birds each (five replicates per group, with 120 chicks per pen):

- Control group (C)—environment without musical enrichment.
- Experimental group (M)—environment enriched with relaxation music.

The groups were reared in separate halls partitioned by a wall. In addition, to prevent sound transmission between the groups (experimental and control), the wall was soundproofed with polyurethane foam, which was secured with a board at the height of the chickens to prevent its pecking by birds. The chickens were reared from 1 to 42 days of age under standard conditions in accordance with flock management instructions [30]. They had constant access to water and feed provided ad libitum (commercial feed mixtures appropriate for each stage of rearing).

Chickens from group M were played ambient relaxation music (“Weightless” by Marconi Union; a = 442 Hz) for two hours a day (8:00–10:00) continuously throughout the rearing period (from day 1 to day 42) and for 30 min prior to slaughter. The music was played through speakers placed individually in each pen at a height of approx. 1 m. The volume of the music (sound pressure level; SPL) was checked on the 1st, 10th, 21st, and 35th day of rearing (at the speaker, in the center of the pen, and in the corner). The average energy from the measurement points and from four days of measurement was 72.3 ± 1.8 dB (min. 70.8 dB; max. 74.9 dB; music + environmental sounds). Group C was exposed only to environmental sounds with a sound pressure level of 60.9 ± 3.9 dB (min. 55.5 dB; max. 64.8 dB). A detailed description of the experimental hall setup and chicken rearing procedures is presented in the publication by Ciborowska et al. (2025) [18]. On the 42nd day of rearing, all chickens from both groups were weighed. Then, two individuals with a body weight close to the average weight in a given group were selected from each pen and slaughtered. After slaughter, the chicken carcasses were cooled at +4 °C for 24 h, after which the pectoral muscles were collected for further analysis.

### 2.2. Analyses of Pectoral Muscle Quality

Entire left pectoral muscles from both groups were used to determine drip loss. The entire right pectoral muscles were first used to measure pH (15 min, 1 h, 4 h, 12 h, 24 h) and color parameters. Next, part of the muscle was homogenized using a laboratory grinder with a 3 mm mesh and thoroughly mixed for further laboratory analysis: water holding capacity (WHC), meat color, proximate chemical composition, reduced glutathione (GSH) concentration (48 h after slaughter) and malondialdehyde (MDA) concentration (48 h after slaughter). The unground part of the right pectoral muscle was frozen in two parts at −20 °C for MDA determination 1 and 3 months post-slaughter.

### 2.3. Determination of Drip Loss

The analysis was conducted using left pectoral muscles, which were weighed after dissection. The muscle samples were then transferred to sealed zip-lock bags and stored in a cold room at +4 °C for 24 h. Afterwards, the pectoral muscles were removed from the bags, gently dried and reweighed. The difference between the two weights was calculated and expressed as a percentage of the sample mass.

### 2.4. Determination of Water Holding Capacity (WHC)

The water holding capacity (WHC) was determined according to the method described by Grau & Hamm (1953) [31], modified by Pohja & Ninivarra (1957) [32]. Minced meat weighing approx. 0.3 g was placed on filter paper with a specified sorption capacity (Whatman 1). The filter paper with the sample was positioned between two glass plates and then evenly loaded with a 2 kg weight for 5 min, after which the samples were dried. A planimeter was used to determine the surface areas of the exudate and squeezed meat samples, and then the WHC index was calculated.

### 2.5. Measurement of pH and Determinations of Color Parameters and Proximate Chemical Composition

In the right pectoral muscle, pH measurements were taken 15 min post-slaughter, as well as after refrigerated storage (+4 °C), 1 h, 4 h, 12 h and 24 h post-slaughter, using a CP-401 pH meter equipped with a glass-calomel electrode (Elmetron, Zabrze, Poland). The color parameters of the meat (L*, a*, b*) were also measured both for the whole (non-minced) pectoral muscle from its external, presentation side (visible to the consumer) and for the minced meat samples. The measurements were performed using a CR-410 colorimeter (Konica Minolta, Tokyo, Japan), and the color deviation tolerance (∆E) between the control and experimental groups was subsequently calculated according to the methodology provided in Michalczuk et al. (2024) [33].

The minced meat was also used to determine its proximate chemical composition (water, protein, fat, and collagen content) via a spectrophotometric technique with near-infrared transmission in the range of 850–1050 nm (NIR) (PN-A-82109; PKN, Warsaw, Poland, 2010) [34] according to the methodology posited by Rant et al. (2019) [35]. The samples were scanned using a FoodScanTM analyzer (FOSS Electric, Hillerød, Denmark).

### 2.6. Determination of Glutathione (GSH) Concentration

Glutathione (GSH) concentration was determined spectrophotometrically using the Ellman method according to Matusiewicz et al. (2019) [36] with minor modifications. The material analyzed 48 h after slaughter was stored at +4 °C. Briefly, 0.1 g of pectoral muscle was homogenized in PBS (pH 7.4) and centrifuged. The supernatant was deproteinized with 50% TCA and centrifuged again. Then, 25 µL of deproteinized supernatant was mixed with 200 µL phosphate buffer (pH 8.0) and 25 µL DTNB in a 96-well microplate. After incubation, absorbance was measured at 412 nm using a microplate reader. GSH concentration was calculated from a standard curve prepared with reduced glutathione and expressed as mg GSH/100 g of meat.

### 2.7. Determination of Malondialdehyde (MDA) Concentration

The level of malondialdehyde (MDA) was determined by adapting the methodology described by Kapusta et al. (2018) [37]. The pectoral muscle samples were stored as non-homogenized integral fragments (in three parts intended for analysis at specific time points). Homogenization was performed immediately before MDA determination. The material analyzed 48 h post-slaughter was stored at +4 °C. Samples intended for analyses after 1 and 3 months were stored at −20 °C. Before determination, the samples were thawed at +4 °C for 12 h. The results are expressed as mg MDA/1 kg of meat.

### 2.8. Statistical Analysis

All the statistical analyses were performed using IBM SPSS Statistics software (PS IMAGO PRO 10.0). The data were first tested for normality using the Shapiro–Wilk test, and the homogeneity of variance between groups was assessed with Levene’s test. Two groups (the control [C] and music [M] groups) were compared. Differences between the groups were analyzed using the *t* test for independent samples. In cases where the assumption of homogeneity of variances was violated, Welch’s correction was applied. The results are presented as the means ± standard deviation (SD). Significance levels of *p* ≤ 0.05 and *p* ≤ 0.01 were adopted, indicating statistically significant and highly significant differences, respectively.

The MDA data were analyzed using the repeated measures analysis of variance (ANOVA) within the General Linear Model (GLM) procedure of IBM SPSS Statistics (version 29.0). The model included a between-subjects factor—group C and M—and a within-subjects factor—storage time (0, 1 and 3 months). The assumption of sphericity was verified using Mauchly’s test; if confirmed, the results were reported using uncorrected degrees of freedom. Post hoc comparisons were performed using Bonferroni correction. Differences were considered statistically significant at *p* < 0.05. The results are presented as means ± standard deviation (SD).

## 3. Results

### 3.1. Determination of Drip Loss and Water Holding Capacity (WHC)

The analyzed physicochemical parameters of the pectoral muscles of the broiler chickens revealed significant differences in drip loss, which was lower in group M than in group C (*p* ≤ 0.01). The WHC index did not differ significantly between the groups (*p* > 0.05) (Table 1).

### 3.2. Measurement of pH, Determinations of Color Parameters and Proximate Chemical Composition

**Figure 1:**
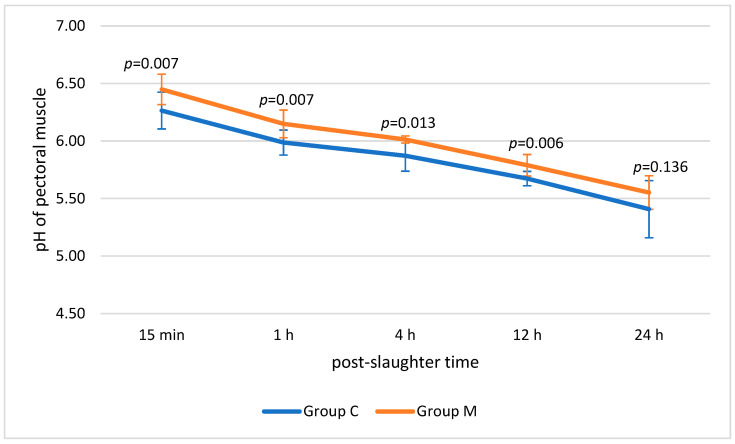
Mean pH of the pectoral muscle of birds from the control (C) and experimental (M) groups (*n* = 10 per group) 15 min, 1 h, 4 h, 12 h and 24 h after slaughter.

The changes in pectoral muscle pH over time are presented in Figure 1. The results revealed statistically significant differences between groups C and M 15 min (6.26 vs. 6.45; *p* ≤ 0.01) and 1 h (5.99 vs. 6.15; *p* ≤ 0.01), as well as after 4 h (5.87 vs. 6.01; *p* ≤ 0.05) and 12 h (5.67 vs. 5.79; *p* ≤ 0.01) post-slaughter. No significant differences were found for the pectoral muscle pH after 24 h (*p* > 0.05). The pH values of muscles from both groups became more acidic; however, the pectoral muscles from group M consistently exhibited a less acidic pH compared to those from group C at each measurement point.

Color measurements of the pectoral muscle were performed for the whole (non-minced) pectoral muscle from its external, presentation side (visible to the consumer) and for minced samples to enable a more detailed assessment of the effects of musical enrichment of the farming environment on these parameters.

Pectoral muscles of the birds from group C had higher values of both the L* and b* color parameters compared to the muscles of group M broilers (*p* ≤ 0.01). In contrast, no significant differences were found for the a* value (*p* > 0.05). The color deviation tolerance (∆E) in the non-minced pectoral muscle was 3.95, indicating a clear color difference visible to the consumer (Table 2).

Statistically significant differences in the L* and b* values were found for minced pectoral meat; group C had a greater L* and b* value than group M (*p* ≤ 0.01). The a* parameter did not differ significantly between the groups (*p* > 0.05). The color deviation tolerance (∆E) for minced muscle reached 5.47, indicating a large and clearly visible color deviation between the groups (Table 3).

The differences in water, protein and fat contents between the groups were small and not statistically significant (*p* > 0.05). However, the meat of chickens in group M had a lower collagen content than that of group C, and the difference was significant (*p* ≤ 0.05) (Table 4).

### 3.3. Determination of Glutathione (GSH) and Malondialdehyde (MDA) Concentration

**Figure 2:**
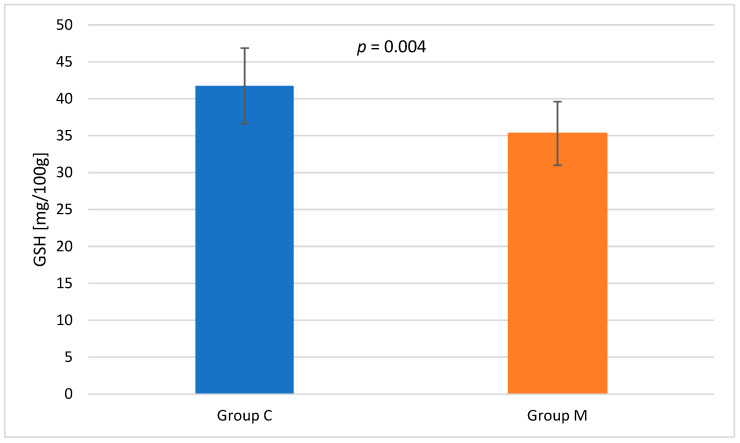
Mean glutathione (GSH) concentration in the pectoral muscle of birds from the control group (C) and experimental group (M) (*n* = 10 per group).

The pectoral muscles of broiler chickens were also analyzed for the concentration of glutathione (GSH) (Figure 2). A significantly higher content of this tripeptide was determined in group C than in group M (41.75 vs. 35.30; *p* ≤ 0.01).

**Figure 3:**
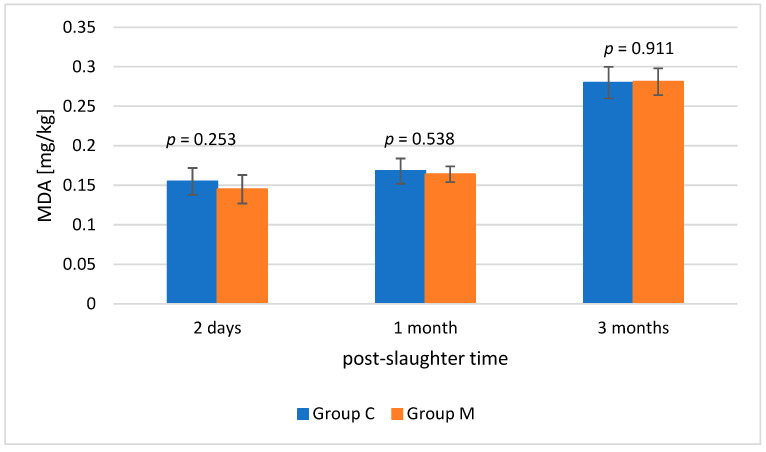
Mean malondialdehyde (MDA) concentration in the pectoral muscle of birds from the control group (C) and experimental group (M) (*n* = 10 per group) 2 days after slaughter, 1 month after slaughter and 3 months after slaughter.

The MDA level increased significantly with extending storage period, from an average of 0.150 mg/kg on the second day post-slaughter to 0.281 mg/kg after three months (*p* ≤ 0.001). The average MDA level was slightly lower in the music-exposed group (0.197 ± 0.003 mg/kg) compared to the control group (0.201 ± 0.003 mg/kg), but the difference was not statistically significant at any time point (*p* > 0.05) (Figure 3). The interaction between time (months) and group was also insignificant (*p* = 0.66), indicating that changes in MDA levels during storage followed a similar trend in both groups.

## 4. Discussion

The quality of broiler chicken pectoral muscles has been extensively discussed for many years. This is due to the increased growth rate of birds and the simultaneous increase in their pectoral muscle size. A common issue in the poultry industry is, among other things, the poor ability of meat to retain water during storage. High drip loss is typically associated with muscle defects such as PSE (pale, soft, exudative) meat [38,39]. In the present experiment, the pectoral muscles of birds from group M were characterized by a significantly lower level of drip loss 24 h post-slaughter compared to those of birds from group C (*p* ≤ 0.05). At the same time, no significant differences were found between the groups in the WHC scores, probably due to the choice of the Grau and Hamm (1953) [31] method, which is based on short-term forced water outflow under pressure and is characterized by a different sensitivity than the drip loss test, as well as greater susceptibility to technical variability. Nevertheless, the trend in water management changes in the analyzed pectoral muscles is consistent. The significantly lesser drip loss determined in group M is probably due to greater protein structure stability after refrigerated storage, despite no significant differences in WHC. Similar results were obtained for drip loss by Berri et al. (2008) [40], who increased the lysine content in the diet of chickens. Similarly, the inclusion of phytogenic additives in the diet of broilers also reduced both the drip loss and WHC values, although the indicators reported in that study were higher than those determined in the present study [33]. Further understanding of the presented results should also be sought in measurements of pectoral muscle pH, which directly affects, among other factors, the aforementioned water-holding capacity and meat color [41].

The pectoral muscles of birds from group C had a lower pH value as early as 15 min after slaughter compared to those of the birds from group M (*p* ≤ 0.01). This trend persisted 1 h (*p* ≤ 0.01), 4 h after slaughter (*p* ≤ 0.05) and 12 h after slaughter (*p* ≤ 0.01) post-slaughter. The measurements made for both groups confirmed the typical changes in meat after slaughter resulting from protein denaturation through lactic acid production [41]. Particularly noteworthy is the low pH_24_ in group C (pH_24_ < 5.5). This value is characteristic of PSE (pale, soft, exudative) meat—further analysis of the results will help explain this value. Tolun and Rathert (2019) [28] also demonstrated the effect of music on increased pH values in broiler meat, although they did not specify the musical genre used in their experiment. A similar initial pH was also obtained in the experiment by Zahoor et al. (2022) [42], where the chickens’ environment was enriched with green and red balls. These authors concluded that this result was due to increased physical activity of the birds and improvements in their welfare. The muscle pH values determined in the present study relate to the water management properties of the pectoral muscle of group M. The higher pH in this group may be associated with the significantly lower drip loss observed, which (despite no significant differences in WHC) suggests better water retention under refrigerated storage conditions and, at the same time, potentially reflects a milder response to pre-slaughter stress. Sun et al. (2019) [43] and Shimokomaki et al. (2017) [44] reported the adverse impact of preslaughter stress on chicken meat quality, including a lower water retention capacity. In addition, Wang et al. (2024) [45] emphasized the importance of long-term musical stimulation in effectively alleviating damage caused by stressors such as noise.

There was a correlation between pH and the color of pectoral muscles, most likely due to the degree of protein denaturation and the directly proportional light scattering. In the muscles with a pH ≥ 6.0, less protein undergoes denaturation than in the muscles with a pH ≤ 6.0; the former may appear darker, and the latter may be lighter [41]. The system developed in 1976 assumes that the L* parameter represents light reflectance (lightness), positive a* values indicate the intensity of the red color, and positive b* values indicate the intensity of the yellow color in meat [46]. In the present experiment, the whole (non-minced) pectoral muscles on the presentation surface (visible to the consumer) and also minced meat samples were analyzed in order to thoroughly assess the impact of relaxing music on color parameters in chicken pectoral muscles. These analyses revealed significant differences between the groups. According to the classification proposed by Lee et al. (2022) [47], the L* value of the pectoral muscles of birds from group M (exposed to relaxing music) was between 56 and 62; hence, their muscles could be considered as of “normal brightness”. In contrast, group C had an L* value greater than 65 for whole (non-minced) pectoral muscles and greater than 63 for minced pectoral muscles, indicating overall paler pectoral muscles. Based on the literature data, it can be concluded that a significantly lower b* value determined in the whole and minced pectoral muscles in group M indicates a lower proportion of yellow color in the meat, whereas a higher value noted in group C indicates its higher contribution in the color profile. According to Yue et al. (2024) [48], meat with a pH < 6.0 is characterized by increased light reflection and thus increased protein denaturation resulting from probable stress. The color parameters determined for the control chickens (group C) confirm the likelihood of the aforementioned occurrence of PSE pectoral muscle defect. The low pH_24_ < 5.5, combined with significantly higher drip loss and high L* and b* values, supports the classification of meat from the control birds (C) as PSE [11]. The reason behind these undesirable changes may be the pre-slaughter stress, which accelerates the metabolic conversion of glycogen into lactic acid, promoting protein denaturation, and ultimately triggering changes in muscle structure, color, and water retention capacity [49]. This confirms the possibility of a positive effect of ambient relaxation music on reducing pre-slaughter stress, which decreased the rate of adverse changes in the pectoral muscle of group M birds. In the study by Gao et al. (2023) [24], classical music from Indian Raga, depending on the stocking density of chickens in the house, did not significantly affect the color parameters of the pectoral muscle; however, according to the authors, the sounds improved the a* value by increasing the antioxidant capacity of the birds’ muscles. A higher color deviation tolerance value was found for the minced pectoral muscles compared to the whole pectoral muscles. This may result from the time elapsed and the oxidative processes that developed between the color measurements of the whole and minced pectoral muscles [50].

Meat quality is also characterized by its proximate chemical composition. The significantly higher collagen content in the pectoral muscles of birds from group C (compared to those from group M) may be associated with texture abnormalities in the meat [51]. Moreover, the lower protein content determined in the same group (although the differences between groups were not statistically significant) supports the assumption of potential textural disturbances in the pectoral muscle. This may indicate a meat defect; however, additional analyses are needed to confirm this finding [52]. Chumngoen and Tan (2015) [53] demonstrated that a higher collagen content in meat is correlated with greater hardness and lower tenderness. Considering the results from the present study (drip loss, WHC, chemical composition) and literature data [51,52,53], the findings suggest a potentially more favorable texture of the pectoral muscle of chickens from group M. Additional direct sensory or instrumental measurements should, however, be performed to confirm this assumption. This is important information for consumers who consider this parameter to be one of the most important sensory attributes when assessing the quality of poultry meat [54]. The impact of music on the chemical composition of meat was also studied by Tolun and Rathert (2019) [28]. However, according to the authors, music resulted in a lower percentage of protein and a higher percentage of crude fat in the meat. Unfortunately, they did not specify the genre of music or the music track used in their study, which makes it impossible to determine the potential causes of the differences between the groups and our study findings.

Important indicators considered in the assessment of oxidative stress include: the concentration of reduced glutathione (GSH) and malondialdehyde (MDA) in the pectoral muscle. GSH provides protective effects against irreversible protein oxidation caused by reactive oxygen species (ROS), which simultaneously attack unsaturated lipids in muscles, causing oxidative damage. The increase in ROS is also accompanied by a directly proportional change in the MDA concentration [55,56]. Bai et al. (2023) [57] reported an increase in GSH levels following glutamine supplementation in chickens exposed to heat stress, which indicates active GSH consumption under oxidative stress conditions. The increase in GSH was accompanied by a reduction in MDA, suggesting a defensive effect of glutamine in the event of an increase in ROS [57]. Similar trends were reported by Skomorucha et al. (2020) [58], who found that the group of chickens supplemented with an herbal mixture in drinking water and having access to a mixture had lower levels of both GSH and MDA. The authors interpreted this finding as a positive phenomenon, indicating a reduced demand for GSH due to lower oxidative stress in birds. In the present experiment, the higher GSH content in group C samples compared to group M (*p* ≤ 0.01) should be interpreted with caution. The result may indicate the mobilization of the antioxidant systems in group C, but it may also result from a smaller antioxidant pool or faster utilization of GSH in group M. Metabolic differences are also likely. The interpretation should be supplemented by the determination of selected antioxidative enzymes—superoxide dismutase (SOD), catalase (CAT), or glutathione peroxidase (GPx), in order to confirm this hypothesis. Unambiguous analysis is also hampered by the fact that the differences in MDA between the groups at selected time points were not statistically significant (*p* > 0.05). MDA values remained at an acceptable level, but there is no evidence to conclude that ambient relaxation music had an effect on lipid peroxidation. Values above 1.0 mg MDA/kg are considered potentially unacceptable [59]. The MDA levels determined after two days of both refrigerated and frozen storage did not exceed this threshold, indicating that the meat from both groups could be classified as of good quality.

In the present experiment, the GSH and MDA values determined for group M, considered together with quality parameters (i.e., drip loss, WHC, pH, meat color, proximate chemical composition), may suggest the modification of the redox balance in the pectoral muscle in response to a musical stimulus to which the birds were exposed during rearing and 30 min prior slaughter. However, additional analyses should be performed to provide a more accurate interpretation of the results regarding the probable reduction in oxidative stress in chickens under the influence of relaxation music. It is worth noting, however, the consistency of the effects of enriching chicken rearing with ambient relaxation music with the results of production parameters and welfare presented in the publication by Ciborowska et al. (2025) [18]. In the cited publication, chickens from the group exposed to ambient relaxation music achieved a significantly higher body weight on day 42 of rearing, with a lower FCR. In addition, the assessment of their welfare and the measurement of stress hormone concentrations in their feces indicated a significant improvement in selected welfare indicators (including footpad dermatitis and gait score) compared to the group not exposed to music [18]. The convergence of these observations with the results obtained in this study may indicate the multi-faceted impact of the selected ambient relaxation music track, but the potential mechanisms of action of this sound stimulus require verification in further research.

## 5. Conclusions

The results of the experiment, which assessed the impact of ambient relaxation music on the quality of pectoral muscles of Ross 308 broiler chickens, revealed positive effects of the music on the following quality parameters: drip loss, pH, L* and b* color parameters (for both whole pectoral muscles and minced meat), collagen content, and GSH concentration. The statistically significant differences demonstrated in the experiment indicate a potential physiological response of chickens to the sounds of the music track, indirectly affecting biochemical processes in their pectoral muscles. This effect is likely related to a reduction in pre-slaughter stress. The trend of changes observed in the experiment aligns with the hypothesized positive effect of the song “Weightless” by Marconi Union, from the ambient relaxation music genre, on the organisms of broiler chickens—this environmental enrichment contributed to the quality of the raw material, namely pectoral muscle. Further research in this area is needed to better understand the mechanism of action of this sound stimulus as an enrichment of the broiler chicken environment.

## Figures and Tables

**Table 1:** Mean drip loss score with water holding capacity (WHC) in the pectoral muscle of birds from the control (C) and experimental (M) groups (*n* = 10 per group).

Parameter	Group	Average	SD ^1^	*p*-Value
Drip loss [%]	C ^2^	0.59 ^A^	0.23	0.001
	M ^3^	0.28 ^B^	0.15	
WHC [g/cm^2^]	C	7.37	2.24	0.201
	M	5.69	3.13	

^1^ SD—standard deviation; ^2^ C—non-treated control group; ^3^ M—group treated with relaxing music; ^A, B^—mean values marked with different letters differ significantly at *p* ≤ 0.01.

**Table 2:** Mean color score for the whole pectoral muscle of birds from the control (C) and experimental (M) groups (*n* = 10 per group).

Parameter	Group	Average	SD ^1^	*p*-Value
L*	C ^2^	65.07 ^A^	2.44	0.006
	M ^3^	61.58 ^B^	2.35	
a*	C	9.51	1.82	0.096
	M	10.61	0.71	
b*	C	19.58 ^A^	0.95	0.005
	M	18.08 ^B^	1.06	
∆E ^4^ C:M	-	3.95	-	-

^1^ SD—standard deviation; ^2^ C—non-treated control group; ^3^ M—group treated with relaxing music; ^4^ ∆E—color deviation tolerance; ^A, B^—mean values marked with different letters differ significantly at *p* ≤ 0.01.

**Table 3:** Mean color score for minced pectoral muscle of birds from the control (C) and experimental (M) groups (*n* = 10 per group).

Parameter	Group	Average	SD ^1^	*p*-Value
L*	C ^2^	63.93 ^A^	3.01	0.006
	M ^3^	58.76 ^B^	4.07	
a*	C	10.32	1.40	0.493
	M	10.74	1.23	
b*	C	19.56 ^A^	1.03	0.007
	M	17.83 ^B^	1.38	
∆E ^4^ C:M	-	5.47	-	-

^1^ SD—standard deviation; ^2^ C—non-treated control group; ^3^ M—group treated with relaxing music; ^4^ ∆E—color deviation tolerance; ^A, B^—mean values marked with different letters differ significantly at *p* ≤ 0.01.

**Table 4:** Proximate chemical composition of the pectoral muscle of birds from the control (C) and experimental (M) groups (*n* = 10 per group).

Parameter	Group	Average	SD ^1^	*p*-Value
Water [%]	C ^2^	74.26	0.70	0.317
	M ^3^	73.82	1.09	
Protein [%]	C	22.01	0.93	0.176
	M	22.44	1.00	
Fat [%]	C	3.01	0.83	0.788
	M	3.10	0.60	
Collagen [%]	C	1.00 ^a^	0.15	0.020
	M	0.83 ^b^	0.13	

^1^ SD—standard deviation; ^2^ C—non-treated control group; ^3^ M—group treated with relaxing music; ^a, b^—mean values marked with different letters differ significantly at *p* ≤ 0.05.

## Data Availability

The data that support the findings of this study are available on request from the corresponding authors (P.C., D.B.).
